# Toward a Better Understanding of Genotype × Environment × Management Interactions—A Global Wheat Initiative Agronomic Research Strategy

**DOI:** 10.3389/fpls.2020.00828

**Published:** 2020-06-16

**Authors:** Brian L. Beres, Jerry L. Hatfield, John A. Kirkegaard, Sanford D. Eigenbrode, William L. Pan, Romulo P. Lollato, James R. Hunt, Sheri Strydhorst, Kenton Porker, Drew Lyon, Joel Ransom, Jochum Wiersma

**Affiliations:** ^1^Lethbridge Research and Development Centre, Prairie Boreal Plain Region, Science and Technology Branch, Agriculture and Agri-Food Canada, Lethbridge, AB, Canada; ^2^USDA-ARS, National Laboratory for Agriculture and the Environment, Ames, IA, United States; ^3^Commonwealth Scientific and Industrial Research Organisation Agriculture and Food, Canberra, ACT, Australia; ^4^Department of Entomology, Plant Pathology and Nematology, College of Agricultural and Life Sciences, University of Idaho, Moscow, ID, United States; ^5^Department of Crop and Soil Sciences, College of Agricultural, Human, and Natural Resource Sciences, Washington State University, Pullman, WA, United States; ^6^Department of Agronomy, College of Agriculture, Kansas State University, Manhattan, KS, United States; ^7^Department of Animal, Plant and Soil Sciences, La Trobe University, Melbourne, VIC, Australia; ^8^Cropping Systems Section, Livestock and Crops Research Branch, Primary Agriculture Division, Alberta Agriculture and Forestry, Barrhead, AB, Canada; ^9^Crop Sciences, Agronomy Group, South Australia Research and Development Institute, Urrbrae, SA, Australia; ^10^Department of Plant Sciences, North Dakota State University, Fargo, ND, United States; ^11^Department of Agronomy and Plant Genetics, University of Minnesota Crookston, Crookston, MN, United States

**Keywords:** wheat, Wheat Initiative, agronomy, Expert Working Group, Genotype × Environment × Management

## Abstract

The Wheat Initiative (WI) and the WI Expert Working Group (EWG) for Agronomy (www.wheatinitiative.org) were formed with a collective goal to “coordinate global wheat research efforts to increase wheat production, quality, and sustainability to advance food security and safety under changing climate conditions.” The Agronomy EWG is responsive to the WI’s research need, “A knowledge exchange strategy to ensure uptake of innovations on farm and to update scientists on changing field realities.” The Agronomy EWG aims to consolidate global expertise for agronomy with a focus on wheat production systems. The overarching approach is to develop and adopt a systems-agronomy framework relevant to any wheat production system. It first establishes the scale of current yield gaps, identifies defensible benchmarks, and takes a holistic approach to understand and overcome exploitable yield gaps to complement genetic increases in potential yield. New opportunities to increase productivity will be sought by exploiting future Genotype × Environment × Management synergies in different wheat systems. To identify research gaps and opportunities for collaboration among different wheat producing regions, the EWG compiled a comprehensive database of currently funded wheat agronomy research (*n* = 782) in countries representing a large proportion of the wheat grown in the world. The yield gap analysis and research database positions the EWG to influence priorities for wheat agronomy research in member countries that would facilitate collaborations, minimize duplication, and maximize the global impact on wheat production systems. This paper outlines a vision for a global WI agronomic research strategy and discusses activities to date. The focus of the WI-EWG is to transform the agronomic research approach in wheat cropping systems, which will be applicable to other crop species.

## Introduction

Genetic improvements in yield continue in the world’s staple crops (Li et al., 2018), but to realize the potential of these improvements in farmer’s fields to meet global demands will require improved agronomic practices (Fischer and Connor, 2018). The yield gap between potential and farm yields for major crops is substantial. For example, farm yields in rice, wheat, and maize are just 80% of potential yields under irrigated conditions, and 50% or less under rainfed conditions (Lobell et al., 2009). Potential yield is defined here as the yield of the best adapted cultivar with current best practice agronomic management ensuring the absence of manageable abiotic and biotic stresses (Fischer, 2015). Potential yields are constrained in many climates by water limitations, but additional constraints are within the capacity of farmers to mitigate. Economic yield is the yield attained by farmers given the prevailing weather, but inputs and practices are applied at the economic optimum (maximizing margin), which may not necessarily coincide with the levels that produce a maximum yield. This remains at approximately 75–85% of potential yield or water limited potential yield (Zhang et al., 2019). The difference between economic yield and farm yield is the exploitable yield gap. Recent research also suggests that the yield gap for the crop sequence is even larger than for individual crops due to inefficiencies in the system as a whole (Hochman et al., 2014). Comprehensive efforts to improve food security must couple genetic increases in potential yield with agronomic approaches to reduce exploitable yield gaps in all major cropping systems. Global intervention to improve agronomy can also increase the resilience of agriculture and agriculture-based livelihoods by increasing and stabilizing the returns to producers and ensuring the capacity of these systems to provide ecosystem services (Food and Agriculture Organization of the United Nations, 2016). Agronomic approaches to achieve these ends can fall under the concept of Agroecology: *The discipline that provides the basic ecological principles for how to study, design, and manage agroecosystems that are both productive and natural resource conserving, and that are culturally sensitive, socially just, and economically viable* (Altieri and Nicholls, 1995).

Wheat is second to rice as a source of calories in developing countries and first as a source of protein (Braun et al., 2010). It is grown on more land area than any other crop. Wheat currently provides 20% of the daily protein and of food calories for 4.5 billion people (Shewry and Hey, 2015). Although estimates of the demand for food by 2050 vary (Hunter et al., 2017), the challenge to increase production by 30–50% is still a major endeavor requiring a global response. Many studies indicate that a warming climate has a general negative effect on yield of staple crops like wheat (Porter et al., 2014). Under projected temperature increases of 2°C above late 20th century levels for the period 2030–2049, models predict wheat yield reductions up to 25% in many areas without modifications of existing cropping practices. However, in some regions, increases in average yield are anticipated due to extended growing seasons and elevated CO_2_. Global temperature increases of approximately 4°C or more would lead to further declines in wheat yield, which when combined with projections of increasing food demand, poses a large risk to regional and global food security.

Wheat production is challenged not only by changes in climate and the extreme growing conditions that could accompany predictions for climate change, but also by changing disease and insect pressures (Figueroa et al., 2018) and management (Zhang et al., 2019). Wheat breeding programs, both public and private, have and will continue to develop new varieties with higher yield potential in the current production conditions and improved resistance to current economic disease and pest problems. While breeding resistance to pest and disease problems is generally thought of as the most cost effective and sustainable approach to combat economic losses, shifts in climate and the resulting changes to weather patterns, cropping system responses, and pest and pathogen populations indicate that breeding targets and goals are likely to shift faster and more frequently than ever before. An integrated deployment of genetics and management options becomes essential in situations where breeders cannot provide genetic solutions in a timely manner or where the frequency of pest and disease outbreaks are too infrequent and variable to allow breeders to incorporate genetic resistance effectively. Furthermore, breeders and government agencies that conduct variety performance evaluations in many of the world’s wheat growing regions test new wheat varieties under relatively constant and often conservative management regimes. The rationale for this approach is quite simple; application of management inputs masks differences in responses to biotic and abiotic stressors among varieties. This means that wheat variety performance evaluations in many regions do not actually measure the attainable yield, but rather the actual yield. This approach potentially biases results in favor of more disease or pest resistant varieties rather than those with the highest genetic yield potential. While logical and defendable, it is a poor reflection of the genetic potential of individual varieties when a cost-effective management input, such a single application of a fungicide, can drastically change the performance and thus ranking of the variety. In many regions, this means that agronomists or producers themselves are left to develop management practices *ad hoc* to exploit the available genotype × management interactions.

Many of the challenges of increasing climate variability, increasing world population, and its resultant impact on food demand and global food security can be addressed by improvements in wheat genetics and agronomy; however, this requires a globally concerted R&D effort. The problem is that these efforts are often fragmented across the globe, conducted in silos, and often lack cross-disciplinary approaches. The recognition of these challenges and issues gave rise to the Wheat Initiative (WI) in 2010^1^. The WI presently has 17 countries as members, two international research centers (International Wheat and Maize Center and International Center for Agricultural Research in Dry Areas), and a number of private sector corporations largely interested in the genetic improvement of wheat (Wheat Initiative, 2020b). There are four themes for research priorities in the WI’s Strategic Research Agenda and two cross-cutting themes that relate to enabling technologies and knowledge sharing and education. Themes 1 and 2 encompass aspects of breeding new wheat cultivars that have increased yield and increased resistance to biotic and abiotic stressors. Theme 3 addresses *protecting the environment and improving the sustainability of wheat production systems.* Theme 4 relates mainly to ensuring the quality and safety of wheat.

Ten Expert Working Groups (EWGs) were established within the WI focused on technical issues, predominantly genetic improvement and crop protection, but also including wheat germplasm conservation, phenotyping, nitrogen use efficiency, and wheat quality (Wheat Initiative, 2020a). In 2016, the WI established a new EWG in Agronomy^2^, recognizing that crop management is an essential complement to genetic improvement in order to achieve the potential economic yields of new and improved wheat cultivars in farmer’s fields. The Agronomy EWG contributes primarily to Theme 3, but also ensures that varieties developed under Themes 1 and 2 approach their potential yield in the hands of farmers. The EWG views itself as a discovery group linking research priorities and research inventories from each country to help improve the efficiency of the global agronomic research efforts in wheat, and to establish synergies among the various agronomy networks around the world. In this policy paper, we describe the vision, aims, and ongoing efforts of the Agronomy EWG within the WI since its establishment in 2016. The EWG acknowledges that certain challenges require an international approach to make significant progress via new collaborative partnerships, more efficient use of research funds, and effective networks to share and communicate new knowledge to farmers who grow wheat in both developed and developing countries. The WI welcomes new members and the EWGs are open for interested parties to join.

## Vision for an Agronomic Research Strategy Within the Wi

The Agronomy EWG will be guided by the principles inherent to sustainable intensification (SI) as called for by the FAO (Hunter et al., 2017) to meet projected increases in demand for food with projected increases in global population. The definition of SI varies, but the Agronomy EWG defines SI as increased agricultural production without adverse environmental impact and without the conversion of additional non-agricultural lands (Hunter et al., 2017). This necessitates increasing farm yields on existing crops lands. While breeders develop new wheat varieties with higher potential yield and resistance to abiotic and biotic stresses, agronomists must design and help implement cropping systems that allow the potential to be realized. These wheat cropping systems must also maintain or improve soil, water, and air quality, and ensure profitability and economic security for farmers. Crucially, those countries that import large quantities of wheat but with potential to increase their domestic wheat production (e.g., countries in Africa, Middle East, and SE Asia) will require agronomists working closely with farmers to develop strategies to adapt new technologies to local conditions more effectively than in the past. By 2030, the Agronomy EWG seeks to promote a framework to improve the effectiveness of global agronomic efforts in wheat-based systems to enhance farm profitability, increase environmental resilience, and ensure an adequate supply of food and feed for the value-added and processing industries.

To accomplish this vision, the focus will be on four priorities with specific actions and outcomes:

### Priority 1—Development of Sustainable Wheat Cropping Systems

Wheat production occurs within a range of different systems worldwide that span the intensive irrigated rice-wheat systems of the Indo-Gangetic Plain, subsidized high input and high yielding systems of northern Europe and China, to the semi-arid broad-acre systems of Australia and North America. Despite the differences in specific details of these systems, most share common challenges including lack of crop diversity, rundown in soil fertility, decreasing terms of trade for growers, increasing risk from climate change, increasing public scrutiny over environmental concerns of soil degradation, N leaching, and chemical usage. Significant research into farming systems innovations such as better integration of legumes and oilseeds, no-till and controlled traffic systems, dual-purpose cropping systems, opportunity cropping to replace summer fallow, and integrated pest, disease and weed management strategies are in progress in many regions of the world. This multi-year systems research provides the broader agronomic framework into which novel wheat genotypes and agronomy must be integrated, and also provide the only way in which to monitor the longer term ecosystem impacts of wheat farming systems such as soil degradation, off-site impacts, and greenhouse gas emissions to inform investigations. As specific new technologies emerge in wheat agronomy (e.g., new autonomous digitally enabled machinery, novel soil microbial amendments, or fertilizer formulations), it will be important to understand how this technology interacts with different systems of production and various growing environments to ensure most effective impact on wheat production systems. The impact of new practices and innovations will be, in part, measured by influences on yield gaps wherever adopted. Yield gaps vary widely depending on country, weather conditions, and soil types; and closing the exploitable gap would add significantly to world wheat production without expansion of current agricultural land. To be useful as a learning and measurement tool, yield gap data need to be developed using a standardized approach that is replicable in different growing regions and accounts for the biophysical limits for production as imposed by weather conditions. Yield gap analysis based on using FAO yield data for wheat collected over time allows the rate of progress in wheat yield to be measured in different countries (Hatfield and Beres, 2019). The ability to calculate yield gaps at a resolution close to the farm level may be possible through the Global Yield Gap Atlas (GYGA) project^3^, which provides a worldview and country differences of actual yields of wheat relative to potential yield and adjusted for weather. The GYGA project also estimates yield gaps at small zones with similar soils and weather, which has utility not only at the local or farm level, but can be upscaled to regional and country levels to contribute to the development of policies and prioritization of research and development funds; and finally to help develop principles for regional cropping system maximizing wheat productivity.

It is recognized that wheat varieties are developed to be adapted to specific growing regions, but wheat production system development is likely to share commonality across growing regions. Partnership in these situations, resulting in large internationally coordinated projects, is a powerful tool to understand the impact and best use of these technologies. The challenge is to have enough understanding of the diverse elements that impact wheat production in order that the best combination of sustainable practices can be employed profitably, so that farmers have the incentive to continue to innovate. The EWG would initially select one or more of the following actions as a pilot to build international partnerships. Workshops held in conjunction with other agronomy scientific conferences would be the primary method to gain active participation and encourage more members from areas currently underrepresented (e.g., Asia, Africa, South America) to join the EWG. Some of the potential collaborative areas are large disciplines that would require further discussion to identify projects with international significance.

#### Actions

• Perform a meta-analysis of research on global wheat production systems.
• Establish wheat yield gaps for all important wheat-producing regions and understand the socio-economic factors that influence yield gap closure.
• Develop diverse wheat cropping systems for improved water and nutrient use efficiency to stabilize and enhance crop production through appropriate choice of crops and agronomic practices in different agro-ecological regions.
• Develop sustainable pest (insects, diseases, weeds) management systems that maintain or build biodiversity.
• Develop cropping systems that reduce greenhouse gases through increased sequestration of CO_2_ and reduced NO emissions.

#### Anticipated Outcomes

• Greater use of legumes (pulses, forages, and cover crops) in wheat crop sequences to reduce reliance on synthetic fertilizers and biocides.
• Increased water and nutrient use efficiency of wheat cropping systems to meet crop demands and reduce damage to the surrounding environment (air, water, soil).
• Reduced greenhouse gas emissions for wheat production systems.

### Priority 2—Improved Management of Wheat Biotic and Abiotic Threats to Sustainable Production

Several agronomy-related issues are common across current wheat systems including the alignment of crop life cycle to changing seasonal patterns; biocide resistance in weeds, fungal pathogens, and insect pests; increasing yields while reducing biocide use due to product withdrawals or social acceptance; provision of sufficient N to achieve potential yields while minimizing environmental damage; new production and product quality possibilities afforded by hybrid, perennial, and gene-edited cultivars of wheat. These themes of research all require linkages between groups (including EWGs) that address single-component issues (e.g., nutrient use efficiency) for effective impact. The Agronomy EWG uses a holistic approach to improve wheat production systems by focusing on the integration of relevant discipline-specific expertise (Hunt et al., 2019). The approach acknowledges the need to capture effective synergies between innovations emerging from discipline-specific research in order to have the greatest impact on production, socio-economic, and ecological outcomes.

#### Actions

• Conduct studies on better aligning crop life cycles, including new wheat varieties with changing seasonal patterns.
• Identify specific management practices for new wheat cultivars to ensure consistent supply and enhanced quality to meet market and consumer needs for feed, food, nutrition, fiber, and other industrial uses.
• Evaluate in-crop non-chemical forms of weed, disease, and insect control to mitigate biocide resistance.
• Develop integrated management systems that allow for lowered use of biocides.
• Development of N management strategies and evaluation of novel products to improve NUE.

#### Anticipated Outcomes

• Scoping studies on possible management interactions with perennial, hybrid, and gene-edited cultivars.
• Improved pest and weed management to reduce the risk of pest outbreaks and employ integrated methods for managing pests.
• Reduction of the carbon footprint in the wheat phase of cropping systems.
• Improved management practices for new wheat cultivars that realize the genetic improvement in potential yield and quality.

### Priority 3—Tools to Support Improved Management Systems for Wheat

Historically, concomitant advancements in breeding and agronomy translated into yield improvements for wheat at the farm level, though the individual contribution of each varied by region (Bell et al., 1995; Nalley et al., 2008; Lollato et al., 2020). Technologies developed to support efforts related to plant breeding more consistently resulted in deployment of tools when compared to agronomy, perhaps because the end user of the tool is the breeder rather than the producer. Fischer and Connor (2018) provide many examples of such technologies, including molecular markers resulting in genomic selection (e.g., Bernardo, 2016), the development of high throughput phenotyping tools (e.g., Araus and Cairns, 2014; Reynolds et al., 2020), and the use of dynamic crop simulation models to inform breeding programs of traits of interest (Chenu et al., 2009; Sciarresi et al., 2019). Despite the potential for improved agronomy through deployment of tools to improve in-season management decisions by producers, these have been scarce.

A few successful examples of tools impacting agronomic on-farm decisions can be cited. The EPIPRE is one of the first interactive decision support systems that incorporated mesoscale weather data and in-field observations to guide producers when to apply fungicides to control foliar pathogens in winter wheat (Zadoks, 1981). A decade later the epidemiological underpinnings of EPIPRE were adapted to the HRSW production of Minnesota and North Dakota^4^. This led to the Fusarium Head Blight Prediction Center that is available to wheat producers across 22 states in the United States^5^. Another example is, Yield Prophet^®^, an internet service that uses a dynamic crop simulation model to inform Australian growers about seasonal yield prospects and potential effects of management practices on yield and profit so that in-season management adjustments were data-driven (Hochman et al., 2009). Tools have also been developed to improve nitrogen management for winter wheat and other crops using remote sensing technologies in the Great Plains region of the United States. These efforts started by estimating the crop’s yield potential using canopy reflectance (Raun et al., 2001), followed by the development of a commercial GreenSeeker^TM^ sensor (Solie et al., 2002), the development of response indices (Mullen et al., 2003), including soil- and seasonal-specific conditions on the crop’s yield potential (Raun et al., 2008). A more recent example includes Canopeo (Patrignani and Ochsner, 2015), an easy-to-use smartphone tool that quantifies fractional green canopy cover and can improve the management of irrigation (Libardi et al., 2019) and wheat grazing in dual-purpose systems (Butchee and Edwards, 2013). While these are successful examples, deployment on-farm is often challenging due to a perception by farm managers or their advisors that adopting new technologies is either too costly or operationally prohibitive (Hochman et al., 2009). Involving growers or other users into the process in a participatory way from the outset would help to overcome issues around on-farm adoption.

The combination of the wealth of agronomic research, the availability of tools to deliver interactive decision support systems, and the nearly ubiquitous access to cellular data networks globally, means that the potential to develop and deploy decision support systems is grossly underutilized. For instance, research papers have exploited the variation in productive capacity for wheat across a field and integrated it with growing season assessments of crop growth for improved nitrogen management (e.g., Schwalbert et al., 2019). Likewise, weed and disease monitoring with remote sensing is an emerging area of crop management (e.g., Franke and Menz, 2007; Cruppe et al., 2017; Pott et al., 2019). Farmers have variable rate seed and fertilizer equipment and often access local weather stations. They also have access to unmanned aerial vehicles (UAVs) that can be used to remotely sense crop growth, the onset of disease and pest stresses, and patterns of water or nutrient stress (Malveaux et al., 2014), though this technology is still at the early stages and opinions on its potential for full integration into on-farm use vary (Freeman and Freeland, 2015). Likewise, no-cost satellite imagery is available to individual producers to help them understand the crop’s yield potential and adjust on-farm decisions accordingly (e.g., Schwalbert et al., 2018, 2020). Precision agriculture has advanced rapidly over the last 20 years (Chlingaryan et al., 2018); however, having more ground-truthing studies is needed to better understand the potential and ROI of proprietary tools around “what works where.”

#### Actions

• Evaluate the efficacy of precision agriculture and large data analysis to improve wheat productivity and sustainability.
• Capitalize on the yield gap assessment as a learning and measurement tool for yield enhancement of wheat.
• Evaluate the ability of images and vegetation indices derived from satellites or ground-based tools to forecast wheat yield mid-season to improve on-farm decisions (e.g., nitrogen management).

#### Anticipated Outcomes

• Increase nutrient use efficiency and reduced environmental impact of over fertilization.
• Improve reliability of the wheat supply through improved yield stability on a regional basis.
• Deployment of new tools to help improve decisions that can contribute to closing the exploitable yield gap.

### Priority 4—Improved Knowledge Mobilization and Sharing

Success improving agronomic practices to enhance wheat productivity and sustainability depends upon designing these practices for compatibility with current technology, cultures, and other regional conditions. Producers face a plethora of risks of which agronomic risks are but one. Consequently, producers often choose risk avoidance strategies even when a scientific body of evidence indicates that particular practices are needed. Success will require farmer consultations and input at the on-set of research projects, rather than after the research is completed. This typically will entail transdisciplinary engagement with farmers and educators in formulating these approaches (Eigenbrode et al., 2018). Using participatory on-farm research networks such as the University of Nebraska On Farm Research Network^6^, or grower groups such as in Australia’s national water-use efficiency Initiative (Kirkegaard et al., 2014), allows not only for validation of small plot research but also identifies early-adopters of new technologies or management tactics who, in turn, can serve as multipliers locally once results have shown to be positive. It will also require effective communication for dissemination to ensure correct implementation and documentation of adoption. A key element to successful adoption is for researchers to recognize and be sensitive to local or regional socio-economic barriers to adoption.

#### Actions

• Strengthen capacity for knowledge sharing through increased membership in the Agronomy EWG (both international and in-country EWGs).
• Assess and implement effective methods of knowledge sharing that are regionally sensitive.
• Adapt a methodology to assess changes in the sustainability of wheat production systems.
• Explore the ‘G × E × M’ × ‘S’ (social aspect).
• Synthesize papers from the GxExM special journal issue into an educational format for use in Continuing Education Credits/Continuing Education Units (CEC/CEU) activities and examinations.
• Develop a metric to determine the degree of exploitable wheat yield gap closure to determine technology sharing success.

#### Anticipated Outcomes

• Increased global linkages and knowledge sharing among producers, agronomists, innovators, scientists, producer organizations, private companies, and governments.
• To better understand how effective knowledge mobilization and sharing systems can be adapted to meet regional needs in a digital world.

## Immediate Goals of the Agronomy Ewg of the Wi

### Goal 1: Develop Agronomic Research Priorities That More Broadly Reflect International Requirements for Collaboration

**FIGURE 1:**
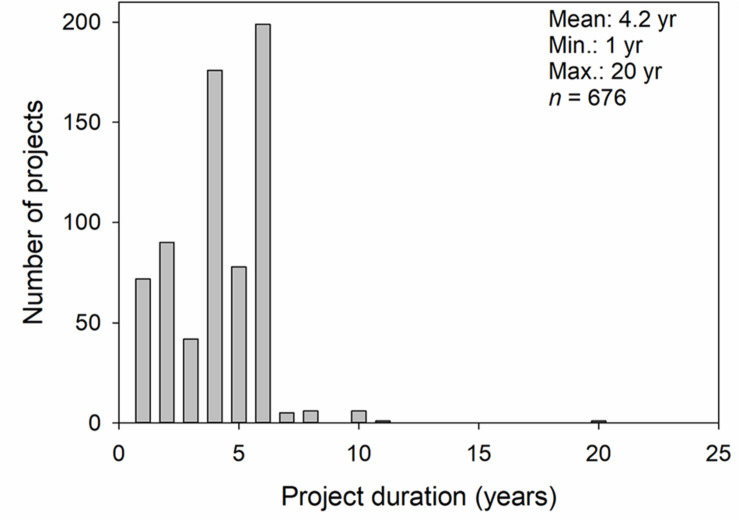
Number of projects in each project duration category for 676 funded wheat agronomy research projects in Australia, Canada, China, Spain, United States, and CIMMYT. A total of 106 projects did not report start or termination dates so project duration was not calculated.

We have taken the first, essential steps toward the development of agronomic research priorities. These included joint meetings of agronomists from different member countries to share information about current projects, and an ongoing effort to establish a database or research inventory of agronomy projects. Beyond the projects led by the agronomists participating in the meetings, we also asked for the participating agronomists to contact colleagues and funding bodies nearby their geography to compile information specifically on wheat that were either funded at the time of data collection (i.e., 2017–18), had recently been terminated, or had received confirmation of future funding. We collected information from 782 research projects originating from Australia (15% of total projects in the database), Canada (30%), China (10%), Spain (1%), United States (42%), and CIMMYT (2%) (Table 1). While 106 projects did not report start or end dates, the start date of the remaining projects ranged from 1999 to 2020; with termination dates ranging from 2015 to 2022. Average project duration (weighted by the number of projects in each duration category) was 4.2 years, ranging from 1 to 20 years (Figure 1). About 30% of the projects were funded for periods of 3 years or less, and the majority (c.a., 67%) of the projects were funded for a period of 4–6 years. The pilot database in its present form is a starting point and will be added to the Wheat Vivo database of the WI and potentially expanded with the inclusion of other key countries that produce wheat (e.g., Argentina, Uruguay, Black Sea Region) and developing countries that might wish to expand wheat production (e.g., North Africa, Sub Sahara, and South Africa). The pilot database has been used by the EWG to analyze ongoing work with collaborative opportunities and perhaps more importantly where there are gaps in knowledge that require a broad approach. While there has been no discussion within the EWG, the database could be further developed into a centralized repository for agronomic data particularly if the EWG is successful in launching internationally coordinated studies to address common research priorities. Data driven agricultural innovation [Genetics, Environment, Management, Socioeconomics (GEMS)], an international effort led by the University of Minnesota (GEMS, 2020) integrates special and temporally distributed genomic, environmental, management, and socio-economic data into a single platform. The interpretation of big data will require a much more diverse expertise than agronomy alone, but offers solutions to real world challenges.

**TABLE 1:** Number of research projects currently funded by Wheat Initiative (WI) Strategic Research Agenda theme and country (or institution).

	**Country or institution**						
**WI strategic research agenda theme**	**Australia**	**Canada**	**China**	**CIMMYT**	**Spain**	**USA**	**Total**
Increase wheat yield potential	6	72	30	4	1	75	188
Controlling wheat diseases and pests	1	43	9	2	–	84	139
Improving wheat tolerance to abiotic stress	2	3	–	1	1	15	22
Nutrient use efficiency	2	10	16	1	4	25	58
Agronomy and crop management	52	72	23	3	1	80	231
Ensure the supply of high quality, safe wheat products	1	24	2	–	–	26	53
Enabling technologies and shared resources	–	10	–	–	–	4	14
Knowledge exchange and education	19	4	–	–	–	18	41
Other	36	–	–	–	–	–	36
Total	119	238	80	11	7	327	782

*The “Other” category included projects that were either transdisciplinary (e.g., interaction between fertility management and weed control), that focused on non-growing season management (e.g., fallow, stubble, or break-crops), or that involved crop modeling and weather-related crop stresses.*

This information is particularly important as longer term funding allows for better sampling of environments, development of probabilistic response curves to given management practices, and for a more representative measurement of sustainability indicators. While single-year funded projects might not allow for development of these indicators and many times consisted of industry-sponsored research protocols; single-year projects can also be extremely relevant in cases where a rapid outcome from agriculture research is needed in response to emerging or unanticipated threats. The EWG will work to grow that database and update it regularly, which will be particularly important as the Agronomy EWG expands its membership.

Initial analysis of the wheat agronomy research inventory showed there is a considerable body of work underway in general agronomy and crop management (30% of the projects in the database), followed by increasing wheat yield potential (24%), and controlling wheat diseases and pests (18%) (Table 1). Other notable projects focused on nutrient use efficiency (7%), ensuring the supply of high quality and safe wheat products (7%), and improving wheat tolerance to abiotic stresses (3%). Only around 5% of the projects were either transdisciplinary, focused on non-growing season management, or involved crop modeling and weather-related crop stresses, and were grouped in the “Other” category. Projects focused on knowledge exchange and education (5%), and enabling technologies and shared resources (2%) completed the reported activities. Much of this work produces regionally specific results with few projects linked to national or international collaborations. However, even though the impacts of the work are often regional and specific to the area in which each project is conducted, many of the constraints they address are universal or at least experienced in some other production systems around the world. While the specifics of agronomy may be context dependent, the principles and approaches of transformational agronomic research can be universal (Hunt et al., 2019). The EWG will work to improve global collaborations addressing common constraints or opportunities and promoting the principles of transformational agronomy. The EWG could play an important role in establishing a central repository for agronomy protocols similar to that put in place by CSIRO in Australia (Nictora and McIntosh, 2011) that would support more standardized procedures as has been evident [e.g., Global Yield Gap Atlas (2020)], and in sharing SI approaches through collaboration and partnerships.

The draft agronomic research strategy developed by the EWG has research priority areas that have been formulated to address the overall need to increase production but balanced to protect the environment. The approach is intended to bring together research, knowledge sharing, and funding capacity to focus on a common vision of SI and outcomes to move the industry forward. An integrated crop management strategy is suggested as the priorities for research, technology sharing, and capacity building are shared while respecting the specific mandates of all contributors. The strategy encompasses crops, soils, environment, climate change, production, and economics. The central assumption is that wheat is not grown in isolation but in systems that include other field crops, the crop/animal interface and the socio-economic context of different production environments. The strategy integrates systems research to: (1) enhance sustainability, both economic and environmental; (2) find more effective methods for long-term crop production, which support and preserve soil, water, air and economic viability of agriculture; (3) enhance economic return through a more efficient conversion of inputs, natural or manmade, to economic product; capturing and holding more components of the system (e.g., carbon credits, biodiversity) and to reduce movement of nutrients beyond the agricultural system (environmental risk); and (4) implement systematic approaches to manage disease, weeds, and insects that are significant threats to crops and the crop/livestock interface that impact value-chains.

### Goal 2: Assessment System for Wheat Sustainability (e.g., an Index); Global Assessment of Wheat Production Systems for Sustainability

A successful global network of sustainable wheat agronomic research will require a common framework of metrics to enable comparative work and sharing of findings, and as a basis to assess the global success of its efforts to reduce yield gaps. The goal would not be to develop a new sustainability index but to understand the work that has been done in many countries and adopting a common approach to measuring sustainability and how SI can be achieved. Adoption of common understanding of definitions and metrics for SI will enable coordinated efforts and serve to amplify the potential impact of the technological advances in wheat production supported by the other EWGs within the WI. A workshop with full participation of the EWG is needed to develop the working definitions for the sustainability of wheat production systems to meet the criteria of SI.

### Goal 3: Country-Specific EWGs for Agronomy Established; Ideas for International Collaborations Developed

To maximize effectiveness, the Agronomy EWG needs nodes for implementation at the country level that bring in key players such as producer groups or associations, private industry, and others such as federal and provincial governments. Thus, performance and success of the Agronomy EWG will also be measurable in terms of level of involvement of key players and development of influential partnerships. The number and types of collaborative partnerships to address the challenges identified by the Wheat Initiative will be key performance indicators. The representational structure that has developed within the Agronomy EWG with members from different regions and countries can be extended to maximize involvement. Agronomy EWG members chosen for their strong credentials, achievements, and influence in wheat agronomy in their countries and regions is needed.

## Wheat Initiative Agronomists Community in the American Society of Agronomy

In 2017, the Agronomy EWG applied to the American Society of Agronomy to consider the formation of a WI Agronomists Community within its Global Section. The main aim of this Community was to consolidate global expertise for agronomy with a focus on wheat production systems. The approach developed and adopted a “systems agronomy framework” relevant to any wheat production system in the world. Such an approach first establishes the scale of current yield gaps identifying physiologically defensible benchmarks, and then takes a holistic approach to understand and overcome exploitable yield gaps. Finally, new opportunities to increase current and potential yield would be sought by capturing future G × E × M synergies identified in different systems. It will be important to have Agronomy EWG in participating countries to feed into the WI as this will allow flexibility for each distinct country and their funding systems.

Supporting aims include:

• Establish a protocol for sharing member country’s approaches and priorities to integrated research, outreach, and policy to improve climate resilience of cereal systems in wheat producing regions worldwide.
• Support interdisciplinary collaboration around wheat production systems through symposia, special publications, and coordinated efforts to link member country funding.
• Expand the scope of the WI to ensure research on wheat is effectively implemented to produce actionable efforts and changes in wheat sustainability, profitability and livelihoods of those dependent on wheat production systems worldwide.

The aim of this Community and the Agronomy EWG is to bring together experts from a broad range of disciplines (vs. a silo approach) who would all contribute to the enhancement of the wheat phase as part of a systems agronomy approach that will meet the global challenges facing wheat growers and end-users today and well into the future. Thus, we encourage members from all supporting disciplines, industry colleagues, policy-makers, and funding stakeholders to join this Community and the WI’s Agronomy EWG.

## Applicability of the Framework for Other Crops

Although the Agronomy EWG within the WI is focused on wheat and wheat-centered systems, its vision and framework as described above is applicable to many other crops grown on a global scale. Advances in genetics and crop breeding can only be realized when deployed in agronomic production systems suited to the natural and social resources of a region and in systems designed with resilience to local biotic and abiotic stresses. We are not aware of initiatives of similar scope for other staple or key nutritional crops. We hope the Agronomy WI will serve as a model and thereby serve more broadly to enhance food security and agricultural sustainability worldwide.

## Author Contributions

BB prepared the original manuscript with input, revisions, and editorial contributions provided by all co-authors. JK, JRH, SE, DL, RL, JW, and SS provided significant contributions in the subsequent revision.

## Conflict of Interest

The authors declare that the research was conducted in the absence of any commercial or financial relationships that could be construed as a potential conflict of interest.

## Footnotes

1 www.wheatinitiative.org

2 https://www.wheatinitiative.org/wheat-agronomy

3 http://www.yieldgap.org

4 https://www.ag.ndsu.edu/cropdisease/small-grain-disease-forecasting-model-homepage

5 http://wheatscab.psu.edu

6 https://cropwatch.unl.edu/on-farm-research
